# The role of group 3 innate lymphoid cell in intestinal disease

**DOI:** 10.3389/fimmu.2023.1171826

**Published:** 2023-04-14

**Authors:** Minghui Li, Zehui Wang, Wei Jiang, Yihan Lu, Jun Zhang

**Affiliations:** Department of Gastroenterology, Nanjing First Hospital, Nanjing Medical University, Nanjing, China

**Keywords:** innate lymphoid cell, intestinal immunity, inflammatory bowel disease, colorectal cancer, immune therapy, plasticity

## Abstract

Group 3 innate lymphoid cells (ILC3s), a novel subpopulation of lymphocytes enriched in the intestinal mucosa, are currently considered as key sentinels in maintaining intestinal immune homeostasis. ILC3s can secrete a series of cytokines such as IL-22 to eliminate intestinal luminal antigens, promote epithelial tissue repair and mucosal barrier integrity, and regulate intestinal immunity by integrating multiple signals from the environment and the host. However, ILC3 dysfunction may be associated with the development and progression of various diseases in the gut. Therefore, in this review, we will discuss the role of ILC3 in intestinal diseases such as enteric infectious diseases, intestinal inflammation, and tumors, with a focus on recent research advances and discoveries to explore potential therapeutic targets.

## Introduction

The importance of the gastrointestinal (GI) tract confers the intestinal immune system to a complex function, allowing tolerance to dietary antigens and commensal microbiota while limiting the transport of harmful pathogens and microorganisms. This mission involves a large number of immune cells, among which ILCs are gaining prominence as abundant resident cells in the intestine. ILCs are a newly defined heterogeneous population of lymphocytes distinct from T and B cells which lack antigen-specific receptors (1). Distributed widely from hematopoietic organs to secondary lymphoid as well as non-lymphoid tissues in the body, ILCs are especially abundant in mucous membranes such as the gastrointestinal tract, airways, and skin, where they sensor and integrate environment signals to rapidly activate the innate immune response, releasing effector molecules that engage mucosal immune defense and tissue homeostasis. Moreover, ILCs respond more immediately after infection or tissue damage and directly activate the subsequent adaptive lymphocytes and myeloid cells *via* co-stimulatory molecules and soluble factors different from adaptive immune cells, which require clonal proliferation and differentiation into specific effector cells after antigen recognition (1, 2). Thus, ILCs have emerged as critical effectors of early immunity.

ILCs were initially divided into three groups in 2013 including ILC1 (which comprises s NK cells and ILC1), ILC2, and ILC3 (3). With a better understanding of the mechanisms of development and the molecules involved in the functions of the T cell and ILC subset, a new classification of ILC was proposed in 2018: cytotoxic NK cell, non-cytotoxic helper ILC1, ILC2, ILC3, and LTi cell (2, 4). Given that ILCs and T cells share similar transcription factors and cytokine production capacities, they are also assumed to be their innate functional counterparts, with NK cells, ILC1, ILC2, and ILC3 representing cytotoxic CD8^+^ T cells, CD4^+^ T helper (Th) cells 1, Th2, and Th17/Th22, respectively (5). ILC1s are characterized by the expression of the T-box transcription factor T-bet (encoded by *Tbx21*) and, in response to IL-12, IL-15, and IL-18 stimulation, generate IFN-γ and TNF to target pathogens such viruses, bacteria, and tumors (6, 7). ILC2s are type 2 immune participants that, when activated by IL-25, IL-33, and thymic stromal lymphopoietin, secrete the type 2 cytokines IL-5 and IL-13 (8). Depending on GATA-binding protein 3 for development, ILC2s are involved in the innate immune response to parasites, allergic inflammation, and the repair of injured tissues by producing amphiregulin (9). ILC3s are the predominant ILC subpopulation in the mice and human intestine, they depend on the expression of the transcription factor RORγt (encoded by *RORC*) for development and produce mainly IL-17 and IL-22 upon activation of local signaling molecules such as IL-23 and IL-1β to coordinate the innate immune response to both intra- and extracellular pathogens and maintain tissue homeostasis (10, 11). Although LTi cells are now classified as a unique ILC subpopulation because they develop from a different precursor distinct from other ILCs, they are still frequently classified as ILC3 for discussion due to their common necessity for RORγt expression and function of the production of IL-17 and IL-22 (2, 12).

The distribution of ILCs in the GI tract is compartmentalized, rising progressively from the oral cavity to the distal GI tract, with the highest density in the ileum and colon, where ILC3 is the major innate lymphocyte population (13). This structural feature predicts an important role for ILC3s in the gut. An increasing number of studies have reported that ILC3s maintain the homeostasis of intestinal immunity by upholding epithelial barrier integrity, promoting lymphoid organ formation, facilitating anti-infective responses, modulating intestinal inflammation, and regulating tumorigenesis and development in various ways (14–16). Therefore, this review will focus on the functions of ILC3 and its multiple roles in intestinal diseases.

## Overview of ILC3

ILC3, the same as T cell and B cell, is also derived from the same common lymphoid progenitor in the bone marrow (17). With the help of several transcription factors, ILC3 gradually differentiates and matures. It is well established that RORγt is necessary for the development of ILC3s because the knockout of RORγt can completely halt ILC3 differentiation, development, and lymph node formation (11, 18). LTi cells were the first ILC3 subset to be recognized. They differentiate from fetal liver cells and play an important role in lymph node and secondary lymphoid tissue formation during embryogenesis by expressing integrin α4β7 and lymphotoxin, and can initiate the formation of cryptopatches and isolated lymphoid follicles in the gut-associated lymphoid tissue after microbial colonization (19, 20).

According to the expression of C-C chemokine receptor type 6(CCR6), ILC3 can be classified into CCR6^+^ILC3s and CCR6^-^ILC3s (21, 22). CCR6^+^ILC3 cells mostly comprise LTi-like cells, which are found primarily in the cryptopatches and isolated lymphoid follicles and are implicated in lymph node composition.

CCR6^-^ILC3 cells are mainly located in the lamina propria and are further divided into two subgroups, NCR^+^ILC3 and NCR^-^ILC3, based on NCR expression, a process that requires the involvement of the transcription factor T-bet. NCR^+^ILC3 accounts for about 70% of intestinal ILC3 and expresses mainly IL-22 and less IL-17, whereas NCR^-^ILC3 accounts for only about 15% of intestinal ILC3 and expresses mainly IL-17 and less IL-22 (23, 24). (**Table 1**) Furthermore, through the upregulation of T-bet, CCR6^-^NCR^-^ ILC3s can further transform into CCR6^-^NCR^+^ ILC3s in the presence of IL-1 and IL-23 (27, 28, 34). This behavior of subgroup transformation has also been reported between ILC subgroups, a property also known as plasticity. For example, ILC3 can be converted to the ILC1 or ex-ILC3 phenotype, which promotes IFN-γ expression and reduces IL-22 and IL-17 production (32, 35, 36). Plasticity is prominent in ILCs and enables them to dynamically change their phenotypes and functions in the face of changes in the tissue environment to realize the mucosal protection function of tissue resident cells. In addition, ILC3 can secrete IL-2 and granulocyte-macrophage colony-stimulating factor (GM-CSF) to further integrate intestinal microbial signals and immune cells like macrophages and T cells to coordinate intestinal immunity (25, 29).

**Table 1:** The subsets of ILC3 in mouse intestine.

Subsets	Markers	Main distribution	Cytokines	Reference
CCR^+^ILC3 (LTi cell)	RORγt^+^ α4β7^+^ CD4^+/-^ CD90^+^ CD117^+^ CD127^+^ CD3^-^ CCR6^+^	Lymphoid tissue Lamina propria	IL-22 IL-17 GM-CSF	(11, 12, 25, 26)
CCR^-^NCR^+^ ILC3	RORγt^+^ T-bet^+^ CD4^-^ CD90^+^ CD117^+^ CD127^+^ NKp46^+^ CCR6^-^	Lamina propria	IL-22 IFN-γ IL-2 GM-CSF	(25, 27–30)
CCR^-^NCR^-^ ILC3	RORγt^+^ CD4^-^ CD90^+^ CD117^-^ CD127^+^ NKp46^-^ CCR6^-^	Lamina propria	IL-22 IL-17 IFN-γ	(27, 31)
ex-ILC3	RORγt^+-^ CD127^+^ T-bet^+^ NKp46^+^ NK1.1^+-^	Lamina propria	IFN-γ	(32, 33)

## The role of ILC3 in intestinal homeostasis

ILC3 is required for the maintenance of the intestinal epithelial barrier and immune homeostasis. This important role is achieved primarily through the secretion of cytokines such as IL-22 and IL-17 by ILC3. Simultaneously, the interaction network between ILC3 and intestinal flora and adaptive immunity is also critical for intestinal homeostasis.

ILC3 is the primary source of IL-22, which has been widely demonstrated to be a crucial factor in mediating the integrity of the intestinal barrier (37). Belonging to the IL-10 family, IL-22 binds to a heterodimeric receptor composed of IL-22R1 and IL-10R2 chains executing active functions (38). The IL-22R1 chain for the IL-22 receptor is predominantly expressed in the intestinal epithelium, implying that the intestine is extremely responsive to IL-22 (39). Indeed, a growing number of studies have confirmed that the IL-22-IL-22R axis plays an important role in the maintenance of intestinal barrier function and immune homeostasis through involvement in immune defense, intestinal epithelial cell proliferation, injury repair, and glycosylation, among other things (39–42). By activating STAT3 in epithelial cells, IL-22 promotes the expression of mucins involved in the composition of the mucus layer to recover mucus-producing goblet cells and alleviate colonic inflammation, as well as regulates intestinal mucosal wound healing (41). Following epithelial tissue injury, IL-22 acts directly on intestinal stem cells in the crypt to migrate and proliferate and restore intestinal epithelial cells, allowing the intestinal barrier to remain intact (43, 44). IL-22 initiates DNA damage response in epithelial stem cells *via* aryl hydrocarbon receptor (AHR)sensing of genotoxic phytochemicals, preventing malignant transformation and cancer development (45). In addition, L-22 promotes intestinal epithelial fucosyltransferase 2 expression and glycosylation which is important for the growth of intestinal mutualistic commensal species and, as a result, limits the colonization of opportunistic pathogens to protect against intestinal barrier damage (46, 47).

IL-17 is mainly produced by NCR^-^ILC3 and is involved in anti-infective and immune-inflammatory responses by inducing the secretion of cytokines and chemokines to recruit neutrophils to the site of infection (48). IL-17 also regulates the production of antimicrobial peptides that fight pathogenic bacteria, particularly fungi and improves intestinal epithelial tight junction proteins to maintain the integrity of the intestinal barrier (49, 50). Therefore, the neutralization of IL-17 or elimination of IL-17 induces or exacerbates intestinal inflammation in a multitude of mice models of colitis, some of which were related to the reduced expression of IL-22 (51, 52). CD93 is a functional IL-17D receptor expressed on mature ILC3s, and its absence results in impaired IL-22 production by ILC3s and decreased expression of IL-22-dependent antimicrobial peptides (53). These findings demonstrate the reciprocal collaboration and functional redundancy of IL-17 and IL-22 in the maintenance of gut epithelial integrity.

The gut is home to trillions of microbiotas and its enormous metabolite pool, extensively participating in the regulation of the host immune system and coordinating intestinal mucosal immunity. The development and function of ILC3 were found to be dependent on the intestinal commensal flora, as both the amount of ILC3 and the function of IL-22 production were affected in germ-free mice or mice treated with antibiotics (21, 54). Moreover, ILC3 activity is regulated by microbial metabolites. AHR is a receptor for tryptophan metabolites in the gut and an important transcription factor for ILC3 (55, 56). The studies found that the development of ILC3 and the function of IL-22 secretion were significantly inhibited after the specific deletion of AHR in mice, allowing the expansion of commensal segment filamentous bacteria(SFB) to induce the Th17 cells-mediated colitis (55, 57, 58). Previous studies have demonstrated that SFB colonization can induce intestinal epithelial cells to secrete serum amyloid A to promote Th17 cell differentiation through IL-22 produced by ILC3 (59). This implies that intestinal flora metabolites control intestinal mucosal fungal resistance and Th17 cell immune responses *via* the AHR-IL-22 axis in ILC3. Short-chain fatty acids (SCFA) are the major metabolites of carbohydrates, which are widely recognized to regulate immune cell activity and intestinal mucosal homeostasis. It has been reported that the SCFA receptor is highly enriched on the surface of colon ILC3, and its specific genetic deletion significantly impairs the proliferation of ILC3 and the production of IL-22, resulting in decreased defense against *Citrobacter rodentium*(*C. rodentium*) infection and tissue repair capacity after injury (60, 61). Meanwhile, SCFA can promote intestinal CD4^+^ T cells and ILC3s produce IL-22 both *in vitro* and *in vivo* to suppress intestinal infection (62). In addition to direct activation through surface receptors, intestinal microbiota can also indirectly activate ILC3 by stimulating myeloid cells and epithelial cells to secrete cytokines such as IL-23 and IL-1β. Under the stimulation of IL-1β secreted by macrophages dependent on microbial signals, ILC3 can excrete IL-2 and GM-CSF. Both have been shown in mice models to control oral tolerance to dietary antigens and to regulate the number of regulatory T cells (Tregs) to orchestrate intestinal immune homeostasis (25, 29). It has been discovered that GM-CSF positively influences macrophage M1-type polarization to produce ILC3-stimulating factors that encourage IL-22 secretion and the production of epithelial antimicrobial peptides (63). Additionally, GM-CSF can regulate neutrophils to promote antibody production by splenic marginal zone B cells (64). As we all know, GM-CSF is a key determinant of myeloid lineage differentiation and is essential for organizing the optimal function of mononuclear phagocytes in pathogen defense. This evidence suggests that ILC3 can integrate stromal and myeloid signals and serves as a bridge for interaction between immune cells. ILCs also express a range of surface molecules such as co-stimulatory or auxiliary molecules to directly promote cellular interactions with adaptive cells to modulate immune homeostasis (Reviewed extensively else (65)). For example, ILC3 can express major histocompatibility complex (MHC) class II to regulate CD4^+^ T cell responses and T cell-dependent colonic IgA production against commensal antigens (66, 67). It also expresses plasma cell cofactors including BAFF, CD40L, and DLL1 to promote B-cell antibody production (64).

As previously stated, ILC3 can secrete IFN-γ in response to inflammatory factor stimulation, which is also found in the blood and tonsil-derived ILC3s. Current research has shown that ILC3 can acquire cytotoxic activity and even develop into NK cells to secret perforin and granzyme B (68–71). This might be one of the ways it prevents pathogenic infection and malignancies at intestinal mucosa sites. Recent studies have also identified the involvement of neuronal signaling and circadian rhythms in the regulation of IL-22 production by intestinal ILC3, thereby maintaining intestinal defenses (72, 73). These findings further reveal the key role of ILC3 as a gut gatekeeper sensing and integrating environmental signals for immune defense.

To summarize, ILC3 protects the gut by regulating commensal tolerance and fighting pathogenic bacteria, repairing the intestinal epithelium to maintain epithelial barrier integrity, and directly or indirectly communicating with other immune cells to maintain intestinal immunity and homeostasis. However, everything has two sides. In intestinal diseases such as inflammatory bowel disease (IBD) and colorectal cancer (CRC), dysregulation of ILC3 function can have the opposite effect.

## The role of ILC3 in infectious disease

An important way in which ILC3 maintains intestinal homeostasis is by promoting immune defense against intestinal bacterial infection when bacteria breach the intestinal barrier. IL-22 is the most significant immune weapon of ILC3 against intestinal pathogens. In mice models, IL-22 is critical for innate responses against intestinal bacterial pathogens such as *C. rodentium* and *Clostridium difficile*, and it can limit systemic pathogen transmission. During the infection of *C. rodentium*, large amounts of IL-22 produced by RORγt^+^ILC3 exert protective effects by encouraging intestinal epithelial cells to express antimicrobial proteins (74, 75). ILC3 also secretes lymphotoxins that activate dendritic cells to produce IL-23, which in turn promotes IL-22 production by ILC3. This circuit increases the antimicrobial efficacy of IL-22. However, in mice with intact T-cell immunity, the effect of the presence or absence of ILC3 on *C. rodentium* infection became less obvious, seemingly illustrating the complementary role of ILC3 and adaptive immunity (18).In addition, AHR and vitamin D receptors are essential for the anti-infective effects of ILC3. Mice lacking AHR and VDR have impaired ILC3 function, reduced IL-22 secretion, and increased susceptibility to *C. rodentium* (76, 77). Previous studies have found that vitamin D3 alters the intestinal mucosal defense barrier and increases susceptibility to colitis induced by *C. rodentium*. This mechanism was subsequently shown to be associated with vitamin D-mediated IL-22 secretion by ILC3.The role of ILC3 in *Salmonella typhimurium* (*S. typhimurium*) infection, which causes foodborne enteritis in humans, has become elusive. Previous studies have shown that ILC3 can induce the expression of the antimicrobial proteins RegIIIβ and RegIIIγ proteins through the secretion of IL-22 to prevent the infection of *S. typhimurium* (78). However, a recent study reported the opposite conclusion. Specifically, *S. typhimurium* produced IL-22 by activating the TLR5-MyD88-IL-23 signaling pathway in antigen-presenting cells to selectively enhance ILC3 but not T cells and ILC3 deficiency in mice allowing better control of *S. typhimurium* infection. Thus, ILC3 appears to have a pathogenic role (79). Limiting bacterial infection by reducing IL-22 production through induction of ILC3 death appears to be a potential host defense mechanism against *S. typhimurium* infection (79).

In addition, ILC3 plays a role in infections such as *Toxoplasma gondii*, *Helicobacter*, *Candida albicans*, and *rotavirus* (80–83). For example, *Candida albicans* is one of the opportunistic pathogenic fungi that first activates the innate immune system when it attacks the intestinal epithelium and then activates the production of IL-17 by Th17 cells (81). However, in the early stages of infection, IL-17 can also be produced by lymphocyte subsets such as ILC3, γδ-T and “natural” Th17 cells to facilitate the rapid clearance of the pathogen and, and all three compensate for their mutual functions (84). Furthermore, the AHR is also essential for intestinal mucosal fungal resistance and is closely linked to the AHR-IL22 axis as described earlier (57, 58).

Thus, the strategic position of ILC3 in the gut confers an important anti-infectious role, and it has been identified to be a central participant in mediating immunity against various pathogens.

## The role of ILC3 in inflammatory bowel disease

IBD is a group of chronic relapsing inflammatory diseases of the gastrointestinal tract characterized by acute episodes and remission periods, including ulcerative colitis (UC) and Crohn’s disease (CD) (85, 86). Patients with active IBD often present with a reduced mucus layer and increased intestinal permeability leading to increased microbiota colonization and invasion damaging intestinal epithelial cells and further aggravating epithelial barrier disruption. ILC3s have a dual role in the pathogenesis of IBD, manifesting as a loss of the balance between the protective phenotype involved in mucus barrier homeostasis and the inflammatory phenotype that promotes the production of inflammatory cytokines in the context of persistent immune dysregulation and cytokine stimulation.

**Figure 1:**
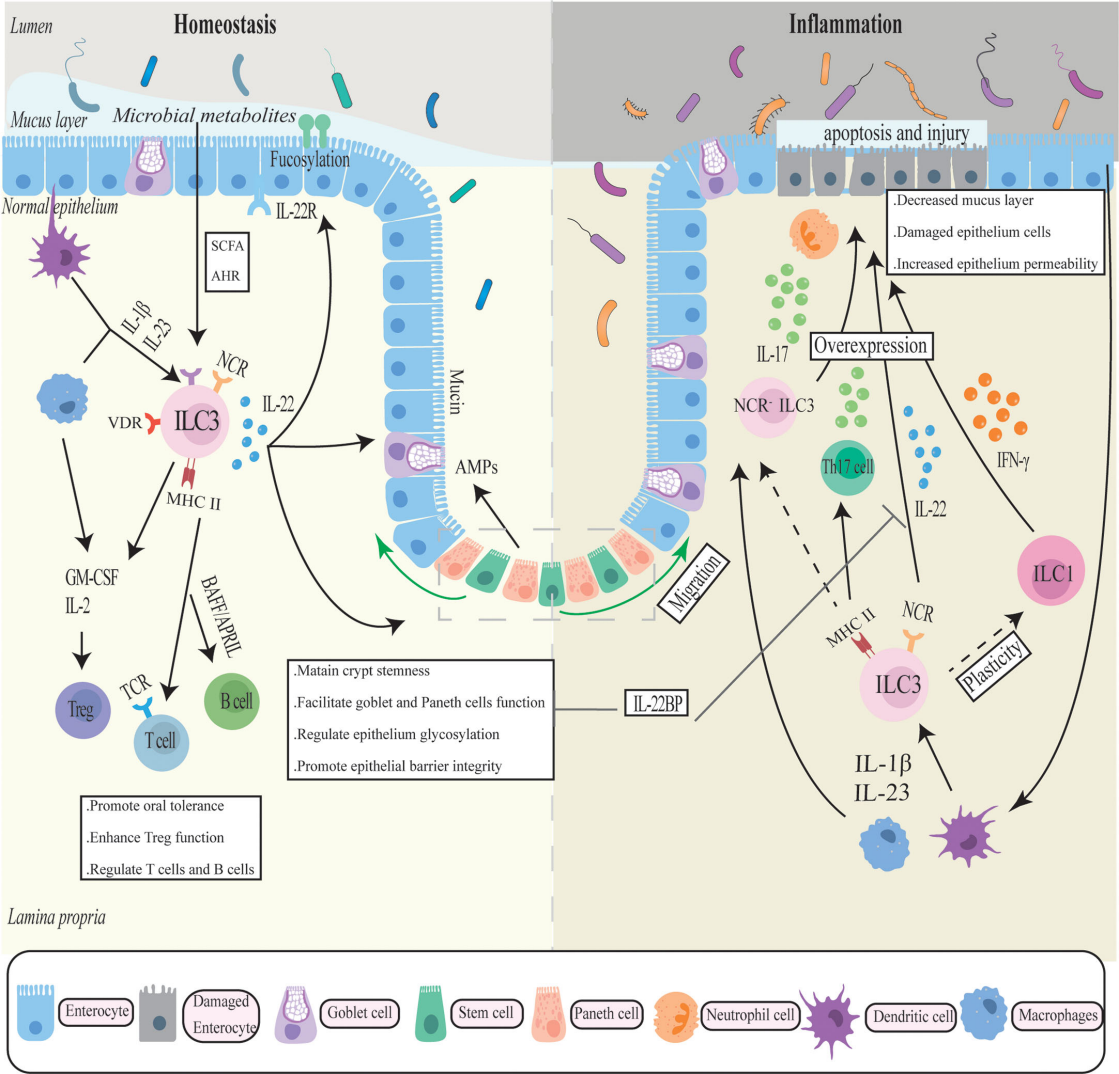
The complicated role of ILC3 in intestinal homeostasis and inflammation. ILC3 generates cytokines such as IL-17, IL-22, IFN-γ, and GM-CSF in response to cytokine stimulation by antigen-presenting cells such as macrophages to maintain the intestinal mucosal barrier. To regulate gut immune, ILC also interacts with a number of immune cells. In chronic inflammation, ILC3 may transfer into a pro-inflammatory phenotype and overexpress inflammatory factors, exacerbating intestinal inflammation and mucosal damage.

As early as 2010, a study reported that ILC3 and chronic intestinal inflammation were associated. The researchers identified a new RORγ+ ILCs population that accumulated in the inflamed colon and secreted IL-22, IFN-γ, and IL-17 in response to IL-23 stimulation, thereby directly mediating IL-23-dependent intestinal inflammation (87). These associations were later expanded in several animal experiments and genetic studies. Multiple ILC3-associated genes (*Rorc, Il22, Il17A, Il17F, Il23r*) have been identified as risk alleles for IBD in genome-wide association studies (GWAS) (88, 89). Even though IL-22 has a demonstrable protective impact on the gut, several studies have revealed that dysregulated IL-22 and other effector molecules released by ILC3 have pro-inflammatory effects in IBD. (**Figure 1**).

In some preclinical IBD models, IL-22 appears to be pathogenic. For example, in anti-CD40 antibody-induced acute innate colitis models, the production of IL-22 derived from ILC3 can cause intestinal inflammation through the stimulation of IL-23 (90). The pathogenicity of IL-22 was also observed in the IL-10 receptor-deficient mice model, where elevated macrophage production of IL-23 induced uncontrolled IL-22 expression in ILC3s in the small intestine, leading to colitis (91). These findings contradict the protective effects of IL-22 in intestinal bacterial infections and may imply a dichotomous role for IL-22. Moreover, IL-22 appears to be associated with autophagy and endoplasmic reticulum (ER)function, both of which are involved in the pathology of IBD. On the one hand, IL -22 synergizing with IL17A can induce ER stress response in the intestinal epithelium related to gut inflammation. And IL-22 deficiency significantly attenuated colitis *in vivo* and *in vitro*, which appears to be associated with reduced levels of ER stress in colonic epithelial tissue. In the absence of ATG16L1, IL-22, on the other hand, facilitated excessive activation of STING-dependent type I interferon signaling, exacerbating intestinal inflammation and cell death (92). ATG16L1 has been identified as an important autophagy gene associated with CD susceptibility that belongs to the mTOR/autophagy pathway (93). Autophagy is a catabolic pathway that maintains intracellular homeostasis. Impairment of the autophagic pathway is associated with an elevated risk of IBD, in patients who tend to develop severe intestinal inflammation, intestinal fibrosis, and stricture. Recent research has discovered that suppressing IL23/IL-22 or depleting ILCs attenuates the fibrotic response in the TNBS-induced fibrosis model. Additionally, the IL23/IL-22 axis was significantly upregulated during fibrosis due to the regulation of the mTOR/autophagy pathway, implying that IL-22 controlled by autophagy signaling was involved in the fibrotic process. Researchers also found that IL-22 can synergistically TGFβ promote the pro-fibrotic response of fibroblasts (94). These new findings further confirm the pathogenic role of IL-22 and provide new insights and feasible targets for the prevention of common fibrotic complications of Crohn’s disease. Moreover, it has been shown that a soluble antagonist of IL-22 binding protein (IL-22BP) was elevated during IBD, which can block the protective biological effects of IL-22 in colitis, particularly mucosal healing (95, 96). TNF was reported to promote IL-22BP expression and lower IL-22 bioavailability, therefore blocking IL-22-driven epithelial cell repair mechanisms in a TNF-driven model of autoimmune colitis (97). Collectively, these findings imply that IL-22 has pathogenic effects that may be related to the activation of endoplasmic reticulum stress and fibrosis, as well as the inhibition of protective effects by autoantagonists.

Various experimental mouse models have shown that the knockdown of IL-17 or blockade of IL-17 expression significantly reverses the degree of colitis, indicating the pathogenic potential of IL-17 (98, 99). Analysis revealed that the main IL-17 producer in colonic inflammatory tissues is NCR^-^ILC3, and the increased amount of NCR^-^ILC3 in CD patients is also accompanied by a reduced frequency of further investigation of innate immune cells isolated from the inflamed colon in the lamina propria of IBD patients revealed elevated expression of ILC3-related genes, with IL-17 exhibiting the highest expression (100). All this evidence supports the idea that intestinal NCR^-^ ILC3 may be involved in the development and progression of intestinal inflammation through the production of IL-17.

In the gut, the expression of MHC II in ILC3 is important for the coordination of intestinal flora and adaptive immunity. A study has reported that selective deletion of MHC II on the surface of ILC3 led to spontaneous intestinal inflammation with bacteria-dependent CD4^+^T cell dysregulation in mouse models (101). Moreover, the expression of MHC II in ILC3 was also identified to orchestrate immunologic tolerance to microbiota and maintain intestinal homeostasis by promoting Treg cells and suppressing Th17 cells. Lyu et al. discovered the destruction of MHC II ILC3 and Treg cells in patients with IBD, and further research found that MHC II ILC3 promoted Treg cells and inhibited pro-inflammatory Th17 cells through antigen presentation, integrin and IL-2 to ordinate microbial immune tolerance and intestinal immunity (102). Compared to non-IBD patients, the expression of MHC II in ILC3 was also reduced in pediatric CD patients, which was linked with Th17 cells that mainly express IL-17A (66). Therefore, the dysfunction of ILC3s may be the root cause of microbial-specific dysregulation of Treg cells and Th17 cells that ultimately lead to IBD.GM-CSF can aggravate intestinal inflammation in mice and humans by inducing additional immune cells such as neutrophils, eosinophils, and monocytes, albeit the ILC3 subpopulations highlighted in these experiments and the mouse models are not identical (30, 103–105). Other molecules including TNF and TL1A also regulate the pathogenic role of ILC3 in intestinal inflammation, and their expression levels are elevated in the intestine of IBD patients. TNF, an important inflammatory factor involved in the pathogenesis of IBD, not only collaborates with IL-23 to initiate the production of pathogenic IL-17 by ILC3s, but also induces chemokine expression that attracts neutrophils, including CXCL1, CXCL2, and CXCL5, which synergizes with IL-17A to further recruit neutrophils to release tissue damage mediators, resulting in exacerbation of intestinal epithelial cell apoptosis and tissue damage (106). TNF-like ligand 1 A (TL1A) and death receptor 3 (DR3) are TNF family ligand-receptor pairs that play a role in IBD pathogenesis. In ILC3s, TL1A increased the expression of the co-stimulatory molecule OX40L, resulting in chronic T-cell-induced colitis (107). DR3 was highly expressed in ILC3s and induced GM-CSF production from ILC3s after activation *via* the p38 MAPK pathway, which drove ILC3 loss from the intestine in an IL-23-dependent manner, as well as colitis deterioration (108). However, previous research has shown that the TL1A/DR3 axis is protective in driving ILC3 IL-22 production and mucosal healing during DSS-induced colitis (107, 109).

In summary, ILC3 has considerable positive effects on intestinal homeostasis on the one hand, but its release of diverse cytokines such as IL-17 and IL-22 may have negative impacts on intestinal inflammation and be influenced by many inflammatory factors on the other. This contradictory role may be related to the tissue microenvironment and inflammatory cytokines in a pathological context, and it may also be influenced by the difference between mice models and human IBD because the former is primarily acute inflammation confined to the intestinal epithelium that can recover rapidly after the exogenous intervention, whereas the latter is a chronic immune disorder-mediated inflammation involving complex multifactorial.

## Potential therapeutic targets in IBD

Immune factors are an important aspect of IBD pathogenesis that has been extensively investigated as therapeutic targets. Early studies mainly focused on the role of adaptive T cells in the pathogenesis of IBD. Nowadays, ILC3 as the innate counterparts of T cells with similar cytokine secretion profiles has gradually attracted attention and has been explored in several preclinical models and clinical models of IBD.

The transcription factor RORγt and the cell surface receptor IL-7R(CD127) are essential for the differentiation of ILC3s and T lymphocytes and have been proposed as therapeutic targets for these pro-inflammatory cells. The blockade of RORγt can inhibit various chronic inflammatory diseases like IBD (110). So far, RORγt inhibitors (GSK805, TAK-828F, and BI119) have shown efficacy in numerous animal models of IBD. Although the above experiments mainly blocked Th17 cells without influencing protective ILC3 responses (111–113). Recently, a novel selective RORγt (VPR-254) inhibitor was shown to reduce the production of key pro-inflammatory cytokines in an Anti-CD40 antibody-induced mouse model of colitis, implying that inhibition of RORγt can also suppress inflammation caused by innate immune-associated cytokines (114). IL-7 and IL-7R levels are not only highly expressed in mucosal tissues of IBD patients, but also overexpression of the IL-7R signaling pathway correlates with the failure of current biological treatments for IBD (115). Further investigations revealed that blocking IL-7R greatly reduced colitis in TRUC mice and humanized mouse models (98, 115). As a result, IL-7R is an important target in the treatment of IBD and a potential biomarker for predicting treatment efficacy.

IL-22, a key effector molecule of ILC3, has been well established for its clinical significance in IBD and is regarded as a promising strategy for IBD therapy. As previously stated, a reduction in IL-22 bioactivity may decrease its protective function in intestinal inflammation and mucosal homeostasis. Thus, using IL-22 fusion proteins to improve IL-22 function appears to be a therapeutic strategy that is effective in the DSS-induced mouse model of colitis (116). The safety and pharmacokinetics of UTTR1147A, the IL-22 recombinant fusion protein, have been studied in healthy volunteers (117). It is also currently being tested in patients with moderate-to-severe UC and CD (NCT02749630, NCT03650413). Given the harmful function of IL-22 in inflammation, IL-22 antagonists have been created and may have a favorable influence on intestinal inflammation by decreasing downstream ER stress and fibrosis. They must, however, be investigated further in IBD in the future (118).

It is worth noting that low-dose IL-2 has been shown to ameliorate colitis in humanized mouse models, which correlates with Treg cell expansion induced by the drug. This revealed its therapeutic potential in IBD and is currently being tested in clinical trials in patients with moderate to severe ulcerative colitis (119). Furthermore, in 2014, vedolizumab, a humanized IgG1 monoclonal antibody that specifically antagonizes the adhesion interaction of α4β7 integrin expressed by leucocytes with MAdCAM-1 (mucosal addressin cell adhesion molecule-1) expressed by intestinal vascular endothelial cells and prevents lymphocyte migration from the vasculature to the intestinal mucosa to reduce the local intestinal inflammatory response (120, 121). The ILC3 migration pathway to the intestine is still unknown, but evidence implies that α4β7, which is regulated by retinoic acid, is highly expressed on ILC3 and is the homing receptor required for ILC3 migration to the intestine (122).

## The role of ILC3 in colorectal cancer

**Figure 2:**
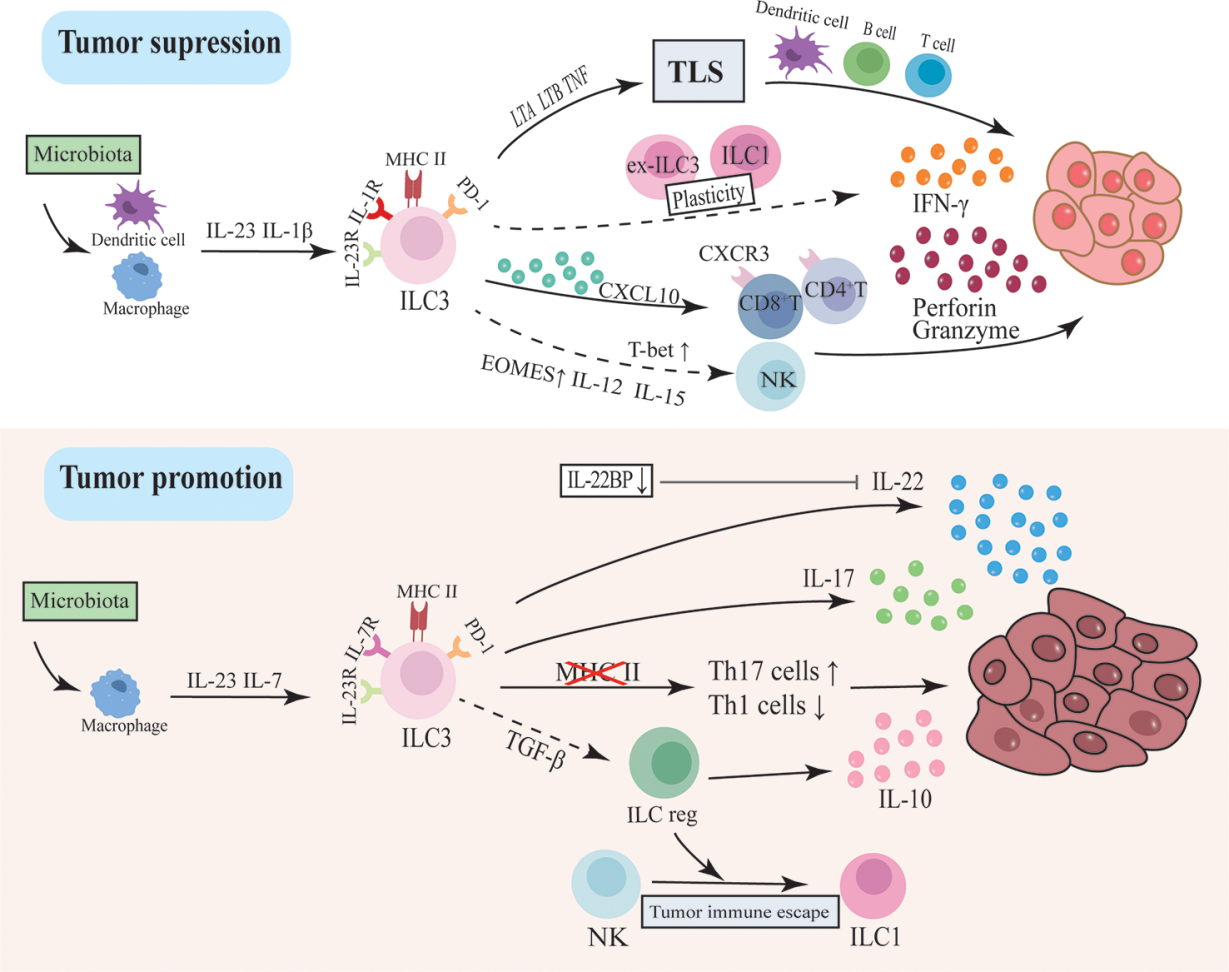
The dual function of ILC3 in intestinal tumorigenesis. ILC3s drive tumor development through inflammatory factors like IL-17 and IL-22. This pathway may be regulated by intestinal flora activating macrophages to secrete IL-23 and IL-7. In addition, the loss of MHC II in ILC3 will lead to dysregulation between Th17 cells and type I immunity, thereby promoting tumor progression. ILC3 can also differentiate into ILCregs and secrete IL-10 to promote tumors. However, ILC3 may inhibit tumor progression by modulating the immune and microbial environment and supporting lymphoid tissue structure. ILC3 secretes CXCL10 and recruits CD4T and CD8T cells to infiltrate tumor cells and secrete IFN-γ, granzyme and perforin to inhibit tumor cells. The plasticity of ILC3 between ILC1 and ex-ILC3 promotes IFN-γ production. The recently discovered ability of ILC3 to differentiate into NK cells may also be an anti-tumor pathway.

CRC is the third most prevalent cancer in humans and the second leading cause of cancer-related deaths worldwide (123). Increasing reports have revealed the role of ILC3s in a range of cancers (124, 125). Tumor-infiltrating lymphocytes (TILs) are lymphocytes including NK cells and cytotoxic T lymphocytes that migrate into the tumor at the time of cancer development and attack the tumor through producing large amounts of IFN-γ or directly killing transformed cells, which is essential for early recognition and clearance of tumor cells. Recently, single-cell RNA sequencing revealed multiple subpopulations of tumor-infiltrating ILC in CRC, as well as their variability and heterogeneity in tumors. ILC3 is altered in CRC and is associated with tumor development and efficacy responses of immunotherapy (126). Immune checkpoints (IC) are a group of molecules that regulate the degree of immune activation including programmed cell death 1 (PD-1)/ligand (PD-L1), and cytotoxic T-lymphocyte antigen-4 (CTLA-4). When activated by immunomodulatory ligands expressed on tumor cells, they can deactivate immune cell activity and cause tumor immune escape and progression. Therefore, the treatment targeted ICs also called immune checkpoint blockade (ICB) has been widely investigated and ultimately achieved revolutionary success by dramatically evoking a long-lasting anti-tumor immune response and being approved by FDA for numerous cancer indications (113). However, this therapy still meets many challenges due to differences in tumor heterogeneity and individual reactivity (113, 114). Noteworthily, ICs have also been expressed in ILCs and blocking PD-1 in ILC2 has shown an enhanced anti-melanoma immune response (127). The functional PD-1 expression has also been detected in human decidua ILC3 and is involved in the regulation of cytokines and immune tolerance (128). Although the study of ILCs and cancer is still in its infancy, recent studies have shown that the co-expression of receptors on ILCs opens up new avenues for cancer treatment. The regulation of ICB expression, in particular, has implications for ICB-insensitive tumors like CRC. The most recent research advances in the pathogenic mechanism and therapeutic response of ILC3 and CRC are discussed in this part. (**Figure 2**)

## Pro-tumor effects of ILC3

Current evidence revealed that ILC3s may drive tumor development through IL-17 and IL-22. Early investigations discovered that IL-22 polymorphisms were related to an increased risk of developing CRC, which was later confirmed in human CRC studies that IL-22 was significantly expressed in tumor tissue and associated with chemoresistance responses (129). In multiple experimental mouse models including CAC, IL-22 secreted by ILC3 has been shown to contribute to tumor development through downstream STAT3 phosphorylation and activation (130–132). IL-7 was recently identified as a cytokine produced by macrophages in response to fungal stimulation, which increased the expression of *Stat3 *and *AhR* in ILC3 to enhance IL‐22 production, eventually promoting the formation of intestinal tumors (132). Because IL-22 also mediates intestinal epithelial tissue regeneration, it is speculated that its overexpression or dysregulation may be responsible for the development of intestinal tumors. Furthermore, the inflammasome-induced downregulation of IL-22 endogenous regulators IL-22BP resulted in uncontrolled IL-22 and promoted inflammation-associated colon cancer progression (133). IL-22BP has been recently shown to decrease in human CRC tissues and have a poorer prognosis. And the antitumorigenic effect of lymphotoxin in mice CRC models is also mediated by increasing the expression of IL-22BP (134).

Multiple animal models have shown that IL-17-driven adaptive and innate immunity contribute to colorectal tumor formation. Intestinal flora and IL-23 are key upstream signals and regulators in this process, and IL-23 can even alone activate ILC3s to induce *de novo* carcinogen-independent intestinal carcinogenesis. It was shown that toll-like receptors identify intestinal flora and subsequently activate downstream nuclear factor kappa B *via* a MyD88-dependent signaling pathway, thereby enhancing IL-23/IL-17 axis expression and causing cancer (135–137). Besides, IL-17 acts on stromal cells to induce various angiogenic factors involved in tumor angiogenesis, as well as to induce the mobilization and recruitment of immature bone myeloid cells resulting in reduced efficacy of anti-angiogenic therapy (138, 139). T cell-derived IL-17 is now being investigated the most intensively, and recent research revealed that inhibiting IL-17 can greatly enhance the expression of IC markers. This was also confirmed in another research, where anti-IL-17A antibodies improved anti-PD-L1 immunotherapy in microsatellite stable (MSS) CRC models (140, 141). Since MSS causes the majority of CRC patients to respond poorly to ICB therapy, this finding suggests that IL-17A could be used as a therapeutic target to sensitize MSS CRC patients to ICI therapy.

The plastic differentiation between ILC3 and ILC1/ex-ILC3 may occur in CRC tissues, where the number of IL-22-producing ILC3 was reduced and IFN-γ-producing ILC1 was increased compared to normal intestinal tissues (142–144). Elevated ILC1 levels have also been found in the peripheral blood of patients with metastatic colon cancer (145). IFN-γ is an important product of ILC1s and has been identified as a critical cytokine in tumor immunosurveillance, whereas IL-22 is a potential tumor-promoting factor (129). However, another study has reported that the number of ILC1 and the production of IFN-γ were significantly suppressed in CRC, and importantly, ILC3s differentiated toward another IL-10-producing regulatory ILCs (ILCregs) under the regulation of TGF-β signaling, ultimately promoting colon tumor progression (146). ILCregs were first identified by Wang et al. in the gut of mice and humans, and they characteristically express Id3 and produce IL-10 to regulate the innate inflammatory response, which is distinct from either ILCs or Treg cells (147). However, Bando et al. found no evidence of ILCregs in the mouse gut while Morita et al. identified that ILCregs originated from ILC2s with the RA stimulation in human nasal tissue (148, 149). Thus, the existence of Id3 ILCregs is controversial and more evidence is still needed to estimate its existence and function.

This evidence revealed the complexity of the plasticity of ILCs and the function of ILC3 in the CRC tumor microenvironment (TME). Many studies have demonstrated that the role of ILC3s in the TME can be sculpted depending on the cancer type, stage, and local tissue environment and ILC plasticity appears to be the result of ILC3-TME crosstalk (150–152).

## Anti-tumor effects of ILC3

ILC3 can also slow tumor progression by modulating the immune and microbial environment and supporting lymphoid tissue structures. Many clinical conventional chemotherapies have immunomodulatory effects and alter the composition and function of TILs, potentially contributing to the ICB immune response (145). Cisplatin is a common chemotherapeutic agent that is being developed in combination with ICB for anticancer treatment in cold tumors. Recent studies found that it may turn cold tumors into hot tumors through ILC3 recruitment of TILs. Cisplatin induces an increase in CCL20 and IL-1β in tumor cells within a cold tumor model, both of which activate ILC3s to release the chemokine CXCL10, attracting CD4 T and CD8 T lymphocytes to infiltrate tumor tissue and then exert anti-tumor effects (153). Similarly, in the MC38 hot tumor mouse model, CCL20 and IL-1β also drove ILC3s infiltration and lymphocytes recruitment to inhibit tumor growth and also enhanced the reactivity to ICB including anti-PD-1 and anti-CTLA-4, significantly extending the survival rate. Furthermore, ILC3 plays an important role in intestinal type 1 immunity and anti-tumor responses by integrating the communication of adaptive immunity and gut microbial tolerance at the intestinal mucosal level. Mice lacking ILC3-specific MHC II were found to develop invasive CRC and resist anti-PD-1 immunotherapy response, which may be associated with the collaboration of increased Th17 cell response and impaired type 1 immunity, implying that MHC II^+^ ILC3 played a protective role in CRC (126). Tertiary lymphatic structures (TLS) are aggregates of TILs at sites of chronic inflammatory stimulation in tumors encompassing B cells, T cells, and dendritic cells. The presence of TLS is considered to be a predictor of favorable outcomes in a variety of malignancies, including CRC (154). The higher the density, degree of infiltration, and maturity of TLS, the better the prognosis of patients. In the human colon, mature NKp44^+^ ILC3 appears to be partially characterized by LTi and expresses the TLS formation-related genes *LTA*, *LTB*, and *TNF*, while in advanced cancers, the expression of the above genes is significantly reduced, followed by a reduction in ILC3, suggesting that ILC3 contributes to protective TLS formation (155). In mice adenoma models, infiltrating ILC3s were also observed to be preferentially positioned inside the TLS, close to T cells with favorable regulation of anti-tumor T cell responses.

ILC3s have recently been displayed to respond directly to tumor cells by enhancing the production of IFN-γ (156). It was discovered that ILC3 may change into an early NK cell phenotype in response to the stimulation of IL-12 and IL-15, owing mostly to the up-regulation of EOMES (68). However, a recent *in vitro* experiment on ILC3 culture by overexpressing T-bet or EOMES found that T-bet was the primary force driving the transformation of ILC3 into mature functional NK cells (69). T-bet and EOMES are recognized to be crucial transcription factors for NK cell differentiation and maturation, and they have a cascade connection with T-bet being more significant towards the end stage. And the cytotoxic ILC3 also have been found in human tonsil tissue (157). Therefore, in the context of different inflammatory factors or transcription factors expression, ILC3 may transition to cytotoxic cells in different directions, and the process may also be a way for ILC3 to participate in the anti-tumor immune response.

The above results reveal some pathways for ILC3 to restrict intestinal tumor progression. It is promising that ILC3 involves in regulating tumor immune cell infiltration and immunotherapeutic response which implies the potential research value of ILC3 in tumor therapy, although the exact mechanisms need to be investigated further in future experiments.

## Potential therapeutic targets in CRC

Based on the discussion above, ILC3 has a dual involvement in the context of intestinal cancer. The relationship between chronic intestinal inflammation and intestinal tumors is becoming increasingly explicit. Many mechanisms are known to promote the development of IBD to CRC, including the NF-B, IL-6/STAT3, and IL-23/IL-17 signaling pathways (158, 159). Thus, inhibiting the activity of pro-inflammatory molecules in the setting of chronic inflammation is another strategy for halting tumor growth. For example, the antagonism of IL-17 and IL-23 has shown anticancer effects in animal models. In the MC38 colon cancer model, IL23R disruption enhanced Treg cells IFN-γ production and recruited CD8^+^ T cells to improve anti-tumor immune responses (160). In addition, blocking TGF-β signaling remarkably suppressed CRC tumor growth by disrupting the differentiation of ILC3s to pro-tumor ILCregs (146). TGF-β has been found to drive the conversion of NK cells into ILC1s to assist tumors in evading surveillance by the innate immune system (161). Thus, inhibiting TGF-signaling in ILC cells may be a viable method to combat cancer.

Despite the fact that immunotherapy was a significant breakthrough in cancer treatment, only a minority of CRC patients respond consistently to ICB (162). But current research has shown that the interplay between ILC3 and T cells is fundamental in inhibiting the course of CRC induced by dysbiosis of the intestinal flora. And ILC3 depletion obviously impaired the effectiveness of anti-PD-1 and anti-CTLA-4 therapy in CRC, indicating that ILC3 operates as an essential protective agent for ICB immunotherapy to exert effective anti-tumor effects (126, 163). In addition to MCH II, ILC3 can regulate T-cell proliferation and function in microbial homeostasis *via* other molecules such as OX40L (153, 164). T cells, as we know, are currently the driving force of CRC immunotherapy, and more T cell infiltration to tumor sites will aid to curb tumor progression. Moreover, TME is further complicated by the involvement of intestinal flora and is associated with resistance to immunotherapy. To summarize, since research on ILC3 and adjuvant ILC in cancer is still in its infant years, on the basis of the latest research achievements, its potential in CRC immunotherapy is promising and prospective.

## Conclusion

ILC3 is a heterogeneous subset of the ILC family that is widely distributed in mucosal sites where they rapidly exert immune defense against foreign pathogens by releasing inflammatory factors or collaborating with other immune cells. It has emerged as the first defensive line in the maintenance of intestinal homeostasis. By regulating intestinal immunity defense, inflammation, and tissue repair, ILC3 also plays a critical role in maintaining the integrity of the intestinal barrier. However, due to their high heterogeneity and plasticity, ILC3s may exert a more complex even pathogenic function in chronic intestinal inflammation and cancer. In the future, a better understanding of the phenotypic and functional characteristics of ILC3, as well as the effects and transitions between ILC3 and other lymphocyte populations in IBD and CRC pathological states, will be required to facilitate understanding of ILC3 function and to identify therapeutic targets for IBD and CRC.

## Author contributions

ML wrote the manuscript and designed the figures. ZW, WJ and YL participated in the framework design of the manuscript and the collection and analysis of the references. JZ critically reviewed the manuscript. All authors contributed to the article and approved the submitted version.
